# Supplementary material for: Three-Dimensional Dendritic Au–Ag Substrate for On-Site SERS Detection of Trace Molecules in Liquid Phase
Source: Nanomaterials (Basel). 2022 Jun 10;12(12):2002. doi: 10.3390/nano12122002 (PMC9229001; doi:10.3390/nano12122002)
Supplement: Supplementary file 1 [file nanomaterials-12-02002-s001.zip › nanomaterials-1733591-supplementary xml.pdf]

nanomaterials

Supplementary Materials

Three-Dimensional Dendritic Au–Ag Substrate for On-Site SERS Detection of Trace Molecules in Liquid Phase

Yunpeng Shao [1,†], Sha Li [1,†], Yue Niu [1], Zezhou Wang [1], Kai Zhang [1], Linyu Mei [1,*] and Yaowu Hao [2,*]

1 School of Mechanical Engineering, North University of China, Taiyuan 030051, China; shypeng@nuc.edu.cn (Y.S.); ls950907@163.com (S.L.); ny057613@163.com (Y.N.); wzz4436@163.com (Z.W.); zhangk950417@gmail.com (K.Z.)
2 Department of Materials Science and Engineering, University of Texas at Arlington, Arlington, TX 76019, USA
* Correspondence: mly81@163.com (L.M.); yhao@uta.edu (Y.H.)
† These authors contributed equally to this work.

Table S1. Main Raman characteristic peaks and band assignments of R6G solution.

Number	Characteristic Peaks（cm^{-1}）	Band Assignments
1	606	the vibration in C-C-C plane
2	764	the bending out of C-H plane
3	1176	the bending in C-H plane
4	1304、1567	the bending vibration mode in N-H plane
5	1356, 1501, 1645	the stretching vibration of aromatic group

Table S2. Main Raman characteristic peaks and band assignments of CIP solution.

Number	Characteristic Peaks（cm^{-1}）	Band Assignments
1	635–695	out-of-plane vibrations of the ring
2	735	methylene swing pattern
3	1023, 1354	C-H oscillation and pyrazine ring mixed vibrations
4	1390	O-C-O symmetric tensile vibration
5	1484	asymmetric stretching vibration of the benzene ring
6	1552	stretching vibration of the quinolone ring system
7	1628	asymmetric stretching vibration of aromatic ring C=O
